# Renal consequences of preterm birth

**DOI:** 10.1186/s40348-016-0068-0

**Published:** 2017-01-18

**Authors:** Amelie Stritzke, Sumesh Thomas, Harish Amin, Christoph Fusch, Abhay Lodha

**Affiliations:** 1 Department of Pediatrics, Section of Neonatology, University of Calgary, Cumming School of Medicine, 780-1403 29th St NW, Calgary, AB T2N 2T9 Canada; 2 Department of Pediatrics, Section of Neonatology, University of Calgary, Cumming School of Medicine, C536-1403 29St Nw, Calgary, AB T2N2T9 Canada; 3 Department of Pediatrics, University of Calgary, C4-615 2888 Shaganappi Trail NW, Calgary, AB T3B 6A8 Canada; 4 Division of Neonatology, Department of Pediatrics, McMaster University, Room 4F5, 1280, Main Street West, Hamilton, Ontario L8S4K1 Canada; 5 Department of Pediatrics, General Hospital, Paracelsus Medical School, South Campus, Breslauer Str. 201, 90471 Nuernberg, Germany; 6 Department of Pediatrics and Community Health Sciences, Alberta Children’s Hospital Research Institute, University of Calgary, C211C 1403 29St NW, Calgary, AB T2N 2T9 Canada

**Keywords:** Developmental origin of health and disease, Fetal origin of adult disease, Renal development, Tubulopathy of prematurity, Prematurity, Kidney disease, Nephrotoxicity, Acute kidney injury

## Abstract

**Background:**

The developmental origin of health and disease concept identifies the brain, cardiovascular, liver, and kidney systems as targets of fetal adverse programming with adult consequences. As the limits of viability in premature infants have been pushed to lower gestational ages, the long-term impact of prematurity on kidneys still remains a significant burden during hospital stay and beyond.

**Objectives:**

The purpose of this study is to summarize available evidence, mechanisms, and short- and long-term renal consequences of prematurity and identify nephroprotective strategies and areas of uncertainty.

**Results:**

Kidney size and nephron number are known to be reduced in surviving premature infants due to disruption of organogenesis at a crucial developmental time point. Inflammation, hyperoxia, and antiangiogenic factors play a role in epigenetic conditioning with potential life-long consequences. Additional kidney injury from hypoperfusion and nephrotoxicity results in structural and functional changes over time which are often unnoticed. Nephropathy of prematurity and acute kidney injury confound glomerular and tubular maturation of preterm kidneys. Kidney protective strategies may ameliorate growth failure and suboptimal neurodevelopmental outcomes in the short term. In later life, subclinical chronic renal disease may progress, even in asymptomatic survivors.

**Conclusion:**

Awareness of renal implications of therapeutic interventions and renal conservation efforts may lead to a variety of short and long-term benefits. Adequate monitoring and supplementation of microelement losses, gathering improved data on renal handling, and exploration of new avenues such as reliable markers of injury and new therapeutic strategies in contemporary populations, as well as long-term follow-up of renal function, is warranted.

## Introduction

Recent advances in medical care and technology have resulted in improved survival of extremely premature infants. Exposure to conditions that lead to preterm birth, premature birth itself, and the management of these fragile neonates may lead to permanent change of organ function and structure. Consequences of alterations in organ function may be more evident in lungs and brain and less evident in other organs, such as the kidney. In keeping with the developmental origin of health and disease (DOHD) concept, survivors of prematurity are at increased risk at later stages of their lives for development of metabolic disease and chronic renal dysfunction [1]. The delay in onset and painlessness of renal disease make recognition and modification difficult but should not deter risk awareness and prudent follow-up.

## Review

### Epigenetic effects

Prevalence rates of premature births are rising due to advanced maternal age, increased use of reproductive technology, and its concomitant increase in multiple gestations [2]. With advances in neonatal care, the survival of preterm infants has substantially improved over the past decades. This has not been consistently mirrored by outcomes in morbidity which remain high, especially in extremely preterm survivors of less than 28 weeks gestational age (GA) at birth [3]. While the pulmonary and neurodevelopmental consequences of prematurity are well under surveillance, the renal effects of prematurity may be less appreciated [4]. Systematic review of over 2 million former low birth weight (LBW) infants concludes an odds ratio of 1.73 to develop chronic renal disease [5]. The National Institute of Child Health and Human Development described the DOHD concept based on Barker’s hypothesis, a concept of developmental plasticity through which a selection of genes are switched on and off in critical periods to adapt the organism to environmental factors [6]. Target organs identified within this concept are the brain, cardiovascular system, liver, and kidney, and its potential life-long impact is recently gathering attention [7].

### Renal development and prematurity

**Fig. 1:**
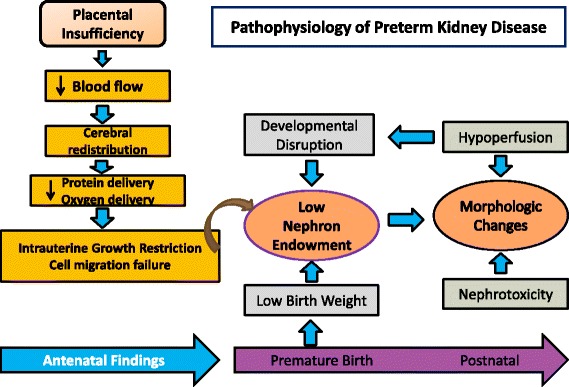
Pathophysiology of preterm kidney disease

At term, there are usually 300,000 to over one million nephrons, a number closely related to birth weight [8]. Nephronogenesis in the human fetus continues until about 34–36 weeks of gestation with more than 60% of nephrons being formed in the last trimester of pregnancy [9]. Organogenesis may be impaired antenatally due to inflammation or intrauterine growth restriction (IUGR) frequently caused by placental insufficiency, resulting in cerebral redistribution and diversion of blood from less vital organs seen via Doppler [10]. Specific antenatal ultrasonographic changes in the kidney are often detectable in these cases with a sausage shape, thought to reflect cell migration failure [11]. In clinical management of high-risk pregnancies affected by these changes, the presence of typical Doppler patterns themselves makes preterm delivery more likely. The very antenatal factors causing prematurity may impact on developmental alterations, with implications caused by prematurity overlapping (Fig. 1).

Prematurity has been a consistently implicated cause for dose-dependent reduction of nephron endowment with lowering GA [12]. A common feature of extremely preterm birth is the disruption of organogenesis and arrest in branching organs. The lungs, vascular tree, and kidney share similar ontogenesis and morphogenesis with branching that normally continues to or past term age. Preterm birth forces the developmental adaptation to an extra-uterine environment with immediate, short-term, and long-term implications. There are similarities in adaptive microstructural changes in these organs with simplification, fibroproliferation, and rarefied, dysmorphic capillaries [13–15].

### Molecular mechanisms of nephron endowment

Molecular pathologic mechanisms implicated in reducing nephron endowment are multifactorial: Poor antenatal perfusion with lack of oxygen and nutrition, in particular protein and micronutrients at a time-critical window for the developing kidney impact nephron numbers [16]. Key molecular influences described perinatally are inflammatory cytokines, reactive oxygen species, and antiangiogenic factors. Inflammation is often thought to be causative in prematurity, and its indicators are associated with later cardiovascular disease [17]. Reactive oxygen species inevitably are generated due to the relative hyperoxia after preterm delivery compared to fetal oxygen tension [18]. Further exposure to oxygen radicals such as from parenteral nutrition, medications, plastics, and X-rays may overcome the immature antioxidant system of the neonate [18]. Increased hypoxia-induced-factor 1 (HIF-1), reduced vascular endothelial growth factor (VEGF) signaling, as well as neonatal endothelial progenitor cells (EPC) being more susceptible to relative hyperoxia result in vessel paucity due to arrest of proliferation and increased apoptosis [19]. There is also increased vessel constriction due to impaired endothelium-mediated vasodilation with oxygen exposure [19]. Furthermore, experimental exposure to hyperoxia for 7 days during postnatal nephrogenesis in mice resulted in a 25% reduction of nephron numbers that persisted into adulthood [20].

Antiangiogenic factors such as endoglin and tyrosine kinase have been shown to be elevated in proportion to the degree of prematurity and ensuing hypertension [14]. The resultant capillary rarefaction and smaller vessel diameter result in undervascularized glomerula in relation to the degree of function [19]. There is a loss of nephrogenic zone in favor of accelerated maturation which leads to early termination of glomerulogenesis [19]. Glomerulogenesis was thought to arrest completely after about 40 days following preterm birth [21]. However, there seems to be a maturational effect of kidney function over time with hyperfiltration, in which fewer glomeruli uptake more blood flow in compensation [22]. Histopathologically, there is evidence of continuous but abnormal glomeruli formation, cystic dilatation of the Bowman’s capsule and atrophic glomerular tufts in up to 18% in a primate model [21, 23]. The body’s mechanism to ameliorate oligonephronia is the activation of the renin-angiotensin system (RAS) to increase glomerular filtration rate (GFR) which is a key factor in genetic hypertension, vascular dysfunction, vessel rigidity, and further constriction [24]. Furthermore, since elastin generation and integration into the vessels occur toward the end of pregnancy, enhanced arterial stiffness is observed in survivors of prematurity [25].

### Aspects of renal impairment in preterm children

Renal injury often remains unnoticed even in adults, as symptoms are rarely life-threatening until potentially irrevocable changes have occurred. In the Neonatal Intensive Care Unit (NICU) setting, optimizing cardiorespiratory function takes precedence in therapeutic targeting to improve mortality. It is well recognized that long-term neurodevelopmental outcomes beyond mere survival are critically dependent on nutrition and optimal growth [26]. Protein accretion in turn is dependent on cellular acid-base status, electrolyte homeostasis, and the conservation of micro- and macroelements. Renal function and its influence on all these factors therefore play a crucial role in optimizing short- and long-term outcome of neonates.

### Glomerular function

The cation primarily responsible for regulation of extracellular fluid volume is sodium. Sodium and fluid management in the very preterm infant is particularly challenging in the immediate postnatal period with limited compensatory mechanisms. Extracellular fluid contraction within the first 3–4 days is a physiologic adaptive response to postnatal life by natriuresis and water loss [27]. In the sick or extremely premature neonate, this process is compounded by systemic and respiratory illness, renal immaturity, environment, and a total dependence on parenteral therapy to maintain homeostasis [28]. Total body sodium and other electrolytes, fluid, and acid-base status in this initial period of contraction in preterm infants balances precariously between intake, the amount and composition of intravenous fluid and oral feeding [29], innate reserves due to maternal sodium and the neonate’s conservation efforts, and ongoing insensible and tubular losses, aggravated by drugs such as diuretics, and those with diuretic effects such as caffeine [30].

### Tubulopathy of prematurity

**Fig. 2:**
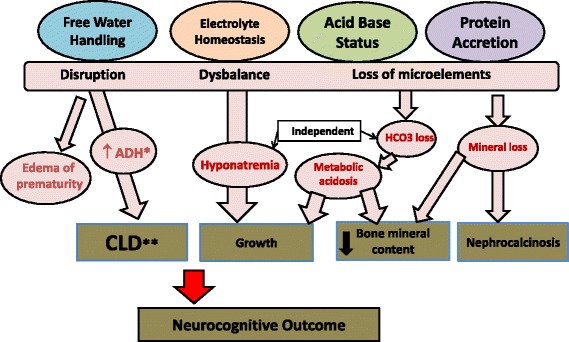
Renal outcomes in prematurity

In older children and adults, renal compensatory mechanisms in pre-renal hypoperfusion states would result in concentration of urine to an osmolality of up to 1000 mOsm/l, urinary sodium concentration of less than 10–20 mEq/l, and fractional excretion of sodium of <1% [31]. However, the maximum urine osmolality in premature infants is about 400 mOsm/l with a fractional excretion of sodium of <4% [32]. A mostly transient condition of “leaky tubules” referred to as *tubulopathy of prematurity* is recognized clinically. It describes a condition of renal immaturity in which, aggravated by limited responsiveness to aldosterone [33], premature kidneys are unable to adequately handle free water, electrolytes, small proteins, and bicarbonate (Fig. 2). This impaired renal concentration ability results in increased free water, which has been implicated in ventilator dependence [34], edema of prematurity, and risk of developing bronchopulmonary dysplasia (BPD) [35]. Loss of bicarbonate, electrolytes, and small proteins may lead to metabolic acidosis, electrolyte imbalance, and poor growth.

Sodium management is particularly challenging with one quarter of infants with GA of less than 33 weeks having a documented episode of hyponatremia of Na <130 mmol/l while in hospital [36]. Inevitably in a number of infants, an evolving sodium deficit remains a challenge to overcome. Supplementation with sodium at a recommended dose of 3–5 mmol/kg/day is generally commenced after weight loss thought to be physiological at around 7% of birth weight has been attained [37], which may not always be achieved, particularly in lower GAs [28]. Monitoring for losses usually occurs as measurement of serum and urine sodium levels. Supplementation is continued in doses aimed at maintaining serum concentration of sodium within an acceptable range (135–145 mmol/l) which in practice often requires amounts well in excess of the recommended dose. The decision how to best supplement is challenging, as serum sodium concentration may be lagging total body sodium depletion and its value is inevitably affected by hydration status [38].

Though easily available, interpretation of preterm urinary sodium loss is difficult, as a high fractional excretion of sodium (FeNa) could signify excess supplementation in functioning tubules, or inability to reabsorb sodium in tubulopathy. Especially in growth-restricted infants, there often is increased combined glomerular permeability of microproteins such as albumin with tubular impairment to reabsorb *N*-acetyl-beta-d-glucosaminidase [39].

### Maturation of renal function in the preterm

With premature delivery, kidney function dynamically changes within the first weeks: The kidney receives 2.5–4% of cardiac output at birth which increases to 15–18% by 6 weeks of life, approaching adult values of 20–25%. The GFR increases from 10–20 to 30–40 ml/min/1.73 m^2^ within days to reach adult values of >75 ml/min/1.73 m^2^ around 2 years of life [40].

Both glomerular and tubular function maturation in the preterm neonate is dependent on GA and postnatal age: Glomerular function is impacted by low initial GFR which increases over time. At day 28, creatinine clearance was still well below term infants’ [39]. There is a high incidence of pathologic proteinuria which may be confounded by immaturity or acute kidney injury (AKI) [39]. However, there is considerable variability in urine albumin and β-2-microglobulins (β2-M) which do not correlate with renal injury markers. Similarly, neutrophil gelatinase-associated lipocalin (NGAL) had been proposed as renal injury marker in prematurity, and does correlate with renal maturity, but not with other injury markers [39]. Urinary cathepsin B activity, a lysosomal tubular proteinase, was proposed as a marker for nephron numbers as it had the strongest inverse correlation with other markers of nephron endowment [39]. There is great capacity for tubular maturation with FeNa approximating term babies at 28 days of life. Protein reabsorption may be lower and slower to recover function compared to electrolyte recovery [22].

### Risks of secondary renal impairment in the preterm infant

Reduced nephron numbers and limited function make impact and consequences of postnatal renal insults greater. Endogenous and iatrogenic factors in the intensive care environment play a role in AKI. Renal failure in premature infants with normal uro-renal morphology may often be subclinical (Fig. 1) and is usually caused by noxious insults such as hypoperfusion and nephrotoxicity which can be classified as pre-renal, or intrinsic. Hypoperfusion is a consequence of cardiovascular decompensation and hypotension, due to a variety of reasons such as asphyxia, blood loss, sepsis or patent ductus arteriosus (PDA). In these settings, the healthy regulation of blood flow via dilatation of the afferent by prostaglandins and vasoconstriction of both efferent and afferent renal vessels by angiotensin is often inhibited resulting in oliguria [40]. Nephrotoxic insults commonly occur from medications, such as antibiotics, antifungals, non-steroidal anti-inflammatory drugs, and diuretics [41]. Despite limited studies to support its use, diuretics are commonly used to reduce ventilatory and oxygen requirements, and methylxanthines like caffeine are ubiquitous, due to the proposed beneficial short and long-term effects on respiratory and developmental status [42]. All of these can be associated with AKI with potentially long-term deleterious effects in summation [43].

### Assessment of renal injury

Assessment of renal function in premature infants and its impairment is difficult and limited. Clinical signs may be late or nonspecific such as olig- or anuria, edema, and electrolyte imbalances with accumulation of nitrogen waste products [38]. Newborns with pre-renal insults or hypoxic-ischemic renal failure are more likely to respond with oliguria/anuria due to the cortical necrosis, infants with nephrotoxic renal insults due to medications are more likely to maintain normal urine output, thus leaving the clinician with limited clinical signs [44]. Serum creatinine is the most commonly used marker of renal function and the gold standard to diagnose AKI. Creatinine is influenced by age, muscle mass, and maturity, as well as maternal creatinine in the first 72 hours of life, after which it slowly increases [45].

Cystatin C is a novel marker which is independent of body composition and size and believed not to be influenced by maternal renal function [46]. Compensatory surface increase and hyperfiltration of fewer glomeruli may affect serum levels of both creatinine and cystatin C [47]; assessment of GFR, however, is superior using cystatin C as a marker [48]. Conclusions about renal endowment based on these markers may be questionable, as it has been shown that effective renal plasma flow is the better marker prognosticating renal outcome [49]. More accessible in the NICU may be clearing pharmacokinetics of common neonatal medications such as aminoglycosides which also approximate effective renal plasma flow [50].

Neonatal definitions for AKI have been recently proposed by Kidney Disease: Improving Global Outcomes (KDIGO) and rely on an acute increase of serum creatinine above baseline to 150–200% (stage 1), 200–300% (stage 2), or >300% or dialysis (stage 3) [51] (Table 1). These represent modifications from the adult AKI definition and are applicable to neonates in the first 120 days of life [40]. With this definition applied, 18% of LBW infants developed some degree of AKI during their hospital stay which was independently associated with an increased mortality of 42% [51]. Reversible alterations of kidney function occur in about 40% of premature infants who are treated with non-steroidal anti-inflammatory agents such as indomethacin for a PDA [40].

**Table 1:** Adapted from KDIGO neonatal AKI definition 2013

	Serum creatinine (μmol/l) rise by	Serum creatinine rise × reference value*	Urinary output (ml/kg/h)
0	< 26.5	< 1.5	≥0.5
1	≥26.5 (48 h)	≥1.5–1.9 (7 days)	<0.5 × 6–12 h
2		≥2–2.9	<0.5 x > 12 h
3	≥221 or dialysis	≥3	<0.3 x ≥ 24 h or anuria x ≥12 h

*Reference creatinine is defined as the lowest previous serum creatinine value

### Hyponatremia, acidosis, and neonatal growth

Low serum or whole body sodium depletion is associated with decreased postnatal growth [52]. Hyponatremia itself has been implicated as a noxious pro-inflammatory condition and an independent risk factor for poor neuromotor outcome at 2 years of age [36]. Somatic growth by cell proliferation is thought to be mediated via a sodium dependent Na/H antiporter system located in the cell wall which increases the action of Na/K ATPase and stimulates growth by alkalinization of the cell interior [53]. With sodium depletion and acidosis, this antiporter system’s activity is diminished and growth failure occurs despite adequate macronutrient intake [54]. Growth of 15 g/kg/day of lean tissue, which is generally considered the target growth for premature babies, requires a net storage of about 1–1.5 mmol/kg/day of sodium to build the extracellular compartment [55]. Suboptimal weight gain in itself is associated with impaired long-term neurodevelopmental outcome [56]. Less than optimal weight gain despite adequate nutritional intake may eventually be found to be one of the markers of tubulopathy (Fig. 2). First shown in surgical neonates, but also in those less than 32 weeks at birth, prophylactic sodium supplementation from 7–35 days of life has achieved improved weight gain without increasing adverse events [57, 58].

Classic late metabolic acidosis of prematurity and inability to adequately excrete acidic equivalents is uncommon with contemporary neonatal care; however, mild metabolic acidosis, i.e., base excess less than minus 4 or bicarbonate less than 18 mmol/l, remains a concern in up to 30% of neonates with *tubulopathy of prematurity* and with use of human milk fortifiers (HMF) [59]. This is often not easily appreciated in some infants due to relative renal compensation for respiratory acidosis associated with BPD as a consequence of very preterm birth. Tachypnea as only manifestation of mild acidosis may go unnoticed or be confounded with mild BPD. There is evidence that the stable growing premature infants with high acid load and age-related low renal capacity to excrete acid exhibits impaired growth and reduced bone mineral content [59].

### Long-term renal consequences

Post-discharge follow-up in premature babies of less than 34 weeks gestation at birth showed a risk of nephrocalcinosis in 14%, associated with nephrotoxic medication use such as dexamethasone, furosemide, theophylline, and aminoglycosides [60]. At follow-up at 2 years of age, survivors with nephrocalcinosis showed impaired tubular function with increased Uca/Ucrea ratio [61]. Although it is not directly associated with systemic hypertension and appears to resolve in the majority of children within a few years, it may persist in 25% up to 7 years of age with unknown life-long consequences [62, 63]. In persistent cases, other reasons for hypercalciuria such as hyperparathyroidism should be excluded.

In survivors of extreme prematurity, renal structural and functional differences were noted: smaller kidney volume and higher cystatin C and blood urea nitrogen (BUN) were detectable in 7- to 11-year-old former extremely low birth weight (ELBW) infants compared to infants who were born at term [64]. Follow-up at 6 years showed that survivors of prematurity less than 33 weeks had similar rates of microalbuminuria, but those with additional AKI also had a lower GFR than those without AKI [65]. Mild renal tubular insufficiency and significantly lower tubular phosphate and bicarbonate reabsorption as well as lower early-morning urine osmolality have been documented up to 7 years of age [63].

In former LBW infants baseline blood pressures were higher, GFRs lower, and salt sensitivity was disproportionally higher, especially in infants with growth restriction, with 47% compared to 18% in term peers [66]. Salt sensitivity, thought to be due to nephron deficit, correlated with ultrasonographic kidney length [66]. There is evidence of microvascular endothelial dysfunction with increased vascular resistance, and reduced vascular diameter which aggravate the effects of nephron deficit in the premature kidney. Increased glomerular capillary pressure distributed over less glomeruli and with less capability of the afferent renal arterioles to adjust incoming pressure results in compensatory glomerulomegaly, hyperfiltration, proteinuria via activation of RAS, and glomerulosclerosis over time [67].

This process can be compensated over many decades until homeostasis can no longer be maintained resulting in premature aging of the kidney [68]. Lifestyle such as increased maternal body mass index (BMI) pre-pregnancy, and excessive weight gain in at-risk populations exacerbate the risk for secondary changes such as arterial hypertension and metabolic syndrome [69]. In term infants, these modifiable risk factors seem to even surpass the effect of birth weight [69]. Secondary focal glomerular sclerosis on top of marginal renal function can lead to progressive chronic kidney disease and ultimately the need for dialysis.

### Conclusions for neonatal care

Renal conservative strategies start right after delivery and continue throughout NICU stay and long-term follow-up with awareness of renal implications of prematurity and avoidance of further injury. Monitoring markers of kidney function such as creatinine, electrolyte, fluid, weight, and acid-base status, careful attention to type and amount of intake, and addressing medication needs with choosing kidney friendly alternatives where possible, as well as monitoring drug levels of renally excreted drugs and reducing prescribed amounts adequately are commonly employed strategies (Table 2). The use of diuretics in oliguria may be a double-edged sword as the increase in urinary output is often offset by an increase in creatinine [70].

**Table 2:** Proposed practice guidelines for optimization of renal conservation efforts

	During NICU	Health Maintenance for survivors of prematurity
Monitoring renal function	Volume status, weight, ins and out Vital signs Serum electrolytes, crea, and FeNa: Supplementation if prudentMaintain target serum electrolyte values	Awareness of prematurity-related increased risk throughout lifespan [7] Assess serially volume status, weight, diuresis Vital signs (esp BP) Tubular parameters (FeNa/β2-M) Glomerular parameters (albumin/creatinine)
Medications	Drug levels/pharmacist input=> Dosing adjustment Taking renal maturation into account Daily evaluation of medications	Awareness of baseline renal function appropriate choice and adjustment of potential medications
Arterial hypertension	Blood pressure monitoring daily as needed in the acute/sick phase or if abnormal Rule out coarctation aortae Consider renal vascular Doppler	Blood pressure measurement With every health maintenance visit Target age-appropriate values [74] Counseling about salt sensitivity
Nephrocalcinosis	Renal ultrasound before discharge	Follow-up ultrasound for resolution If progression consider urine Ca/creatinine

Advancement in renal protective strategies may be in avoidance of edema and fluid overload which seems to be detrimental, with renal replacement therapy. Renal replacement therapy can be considered in refractory acidosis, uremia, electrolyte disbalance, nutritional deficits, and especially fluid overload [71]. The method of choice in the preterm, peritoneal dialysis, may be considered earlier and although there is a paucity of data on efficacy and practicality, it has been employed in neonates as small as 830 g [72]. An important consideration beyond peritoneal dialysis is the availability of continuous renal replacement therapy systems designed for the neonate which accommodate smaller extracorporeal volumes and higher accuracy [73]. This therapeutic modality will likely expand, as centers’ expertise, comfort level and further evidence gathers.

Long-term strategies include the following: Regular search for and documenting presence of hypertension with appropriate workup and choice of medication if necessary [74]; Documenting and following serially the presence of nephrocalcinosis; and Communication to families and health care providers regarding influenceable lifestyle choices such as salt intake and other risk factor for metabolic syndrome in follow-up care.

### Open questions and implications for research

It is important to recognize that the complications of neonatal renal impairment may present significant challenges in the adult future even in asymptomatic survivors of premature birth. Awareness of deleterious side effects and renal consequences of therapies in the modern NICUs and improved post-discharge care and longitudinal renal follow-up as well as parental and health care provider awareness are warranted. Monitoring of neonatal sodium values in serum and total body sodium monitoring with exploration of non-invasive longitudinal markers of sodium loss such as urinary sodium values may be prudent. Gestation-specific parameters in urinary sodium handling may be helpful to assess whole body sodium status non-invasively. Meticulous attention to nutritional support along with supplementation with bicarbonate, sodium, phosphate, and other micronutrients in neonates with increased tubular losses may be beneficial. Improved neonatal growth is associated with better long-term neurodevelopmental outcomes of prematurity [56]. Normative data to assess renal/tubular function in extreme preterm infants will help in the understanding of their unique physiology of postnatal adaptation and growth. Long-term implications of nephrocalcinosis are missing. Novel ways to assess for severity of nephron reduction and AKI such as urinary cathepsin B and NGAL may be expanded upon. More data on consideration of aminophylline to prevent the adenosine-mediated renal vasoconstriction in asphyxia may be gathered [75].

The application of the KDIGO definition of AKI and its implication for outcomes are unanswered questions, as well as novel reno-protective strategies such as avoidance of fluid overload and its impact on neonatal outcomes, as well as the exploration of renal replacement therapy to minimize the trajectorial risk toward chronic kidney disease.

## Conclusions

The toll of prematurity-related renal injury on the probability of kidney disease in adulthood is understudied. Data on renal handling and improved kidney care in contemporary populations is rare. Survivors of extreme prematurity suffer arrested development of organs with reduced nephron endowment as a consequence of hypoxic-ischemic and nephrotoxic renal insults. Short-term consequences include electrolyte disbalances, acidosis, and impaired free water handling. These could potentially result in prolonged respiratory support, growth failure, and suboptimal neurodevelopmental outcomes in the short term.

In later life, subclinical chronic renal insufficiency may progress even in the asymptomatic survivor. For the neonatologist, the new frontier of improving extremely premature infants’ outcomes also depends on the awareness of renal implications of therapeutic interventions and renal conservation strategies with adequate supplementation and prudent follow-up. Novel markers of AKI such as cystatin C, NGAL, and urinary cathepsin, as well as new treatment strategies such as early dialysis can be explored further. Finally, a same language with AKI definitions in the neonatal population and its impact on outcomes should be the focus of interest.

## Abbreviations

ADH: Anti diuretic hormone
AKI: Acute kidney injury
B2-M: Beta-2-microglobulin
BMI: Body mass index
BPD: Bronchopulmonary dysplasia
BUN: Blood urea nitrogen
CPAP: Continuous positive airway pressure
DOHD: Developmental origin of health and disease
ELBW: Extremely low birth weight
EPC: Endothelial progenitor cells
FeNa: Fractional excretion of sodium
GA: Gestational age
GFR: Glomerular filtration rate
HIF-1: Hypoxia-induced factor 1
HMF: Human milk fortifier
IUGR: Intrauterine growth restriction
KDIGO: Kidney Disease: Improving Global Outcomes
LBW: Low birth weight
NGAL: Neutrophil gelatinase associated lipocalin
NICU: Neonatal Intensive Care Unit
PDA: Patent ductus arteriosus
RAS: Renin-angiotensin system
VEGF: Vascular endothelial growth factor
